# Outcomes of Venous-Arterial Extracorporeal Membrane Oxygenation Therapy in Acute Pulmonary Embolism: A Single-Center Experience

**DOI:** 10.1007/s11239-026-03298-0

**Published:** 2026-05-20

**Authors:** Caleb J. Chiang, Laith Alhuneafat, Peter S. Salama, Andrea M. Elliott, Demetris Yannopoulos, Jason A. Bartos, Alejandra Gutierrez Bernal

**Affiliations:** 1 Cardiovascular Division, Department of Medicine, University of Minnesota, 420 Delaware St SE, MMC 508, Minneapolis, MN 55455 USA; 2 University of Minnesota Medical School, 420 Delaware St SE, Minneapolis, MN 55455 USA

**Keywords:** Extracorporeal membrane oxygenation, Pulmonary embolism, Shock, Cardiac arrest

## Abstract

Outcomes of venous-arterial extracorporeal membrane oxygenation (VA-ECMO) therapy in acute pulmonary embolism. High-risk pulmonary embolism (PE) has a high mortality risk. VA-ECMO provides essential cardiopulmonary support, although data outcomes remain limited. We sought to describe the characteristics and clinical outcomes of patients supported with VA-ECMO for the management of high-risk PE. We retrospectively analyzed outcomes of adult patients treated with VA-ECMO for high-risk PE at the University of Minnesota between January 2015 and May 2024. High-risk PE was defined by the European Society of Cardiology criteria. Clinical, demographic, procedural, and outcome data were analyzed. Mortality predictors were assessed using logistic regression. Of 1350 patients supported by VA-ECMO, 58 (4.3%) presented with high-risk PE (69% cardiac arrest, 31% shock). Mortality was significantly higher among patients cannulated for cardiac arrest than for shock (72.5% vs. 33.3%, *P* = 0.005). An independent predictor of mortality was out-of-hospital cardiac arrest (OR 6.67, 95% CI 1.15–38.7, *P* = 0.035). The duration of CPR was shorter among survivors (25.9 vs. 44.2 min, *P* = 0.02). Neither catheter-directed therapy nor systemic thrombolysis was associated with survival. Major bleeding occurred in 67% of patients and was not significantly associated with mortality. Neurologic injury was the leading cause of death in cardiac arrest patients; multiorgan failure predominated in the shock group. Despite the use of VA-ECMO, mortality in high-risk PE remains elevated, but differed by indication for cannulation. Survival was higher in patients presenting with shock, in-hospital cardiac arrest, shorter CPR duration, and sustained return of spontaneous circulation. Further research is needed to define the role of VA-ECMO and adjunctive therapies across varied clinical presentations.

**Graphical Abstract:**

**Figure:**
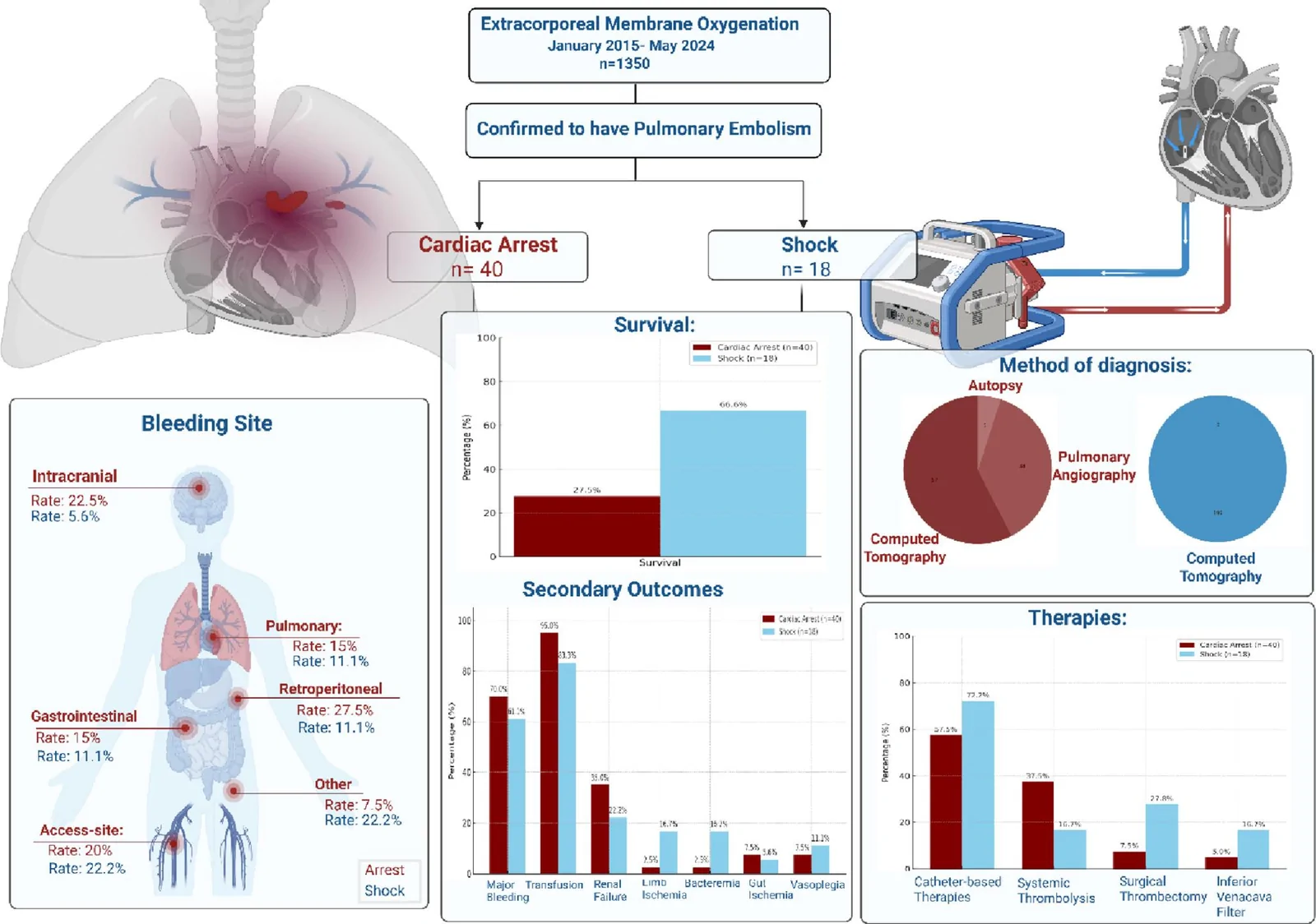
Outcomes of venous-arterial extracorporeal membrane oxygenation therapy in acute pulmonary embolism

**Supplementary Information:**

The online version contains supplementary material available at https://doi.org/10.1007/s11239-026-03298-0.

## Highlights

High-risk PE patients supported with VA-ECMO had high overall mortality, particularly those cannulated following cardiac arrest.

Out-of-hospital cardiac arrest and prolonged CPR were strong predictors of poor outcomes in the cardiac arrest cohort.

Use of catheter-directed therapy or systemic thrombolysis did not demonstrate a survival advantage in either cohort.

Patterns of mortality differed by presentation: neurologic injury in cardiac arrest vs. multiorgan failure in shock.

Future directions: Prospective studies are needed to identify optimal timing, patient selection, and the role of adjunctive reperfusion strategies in VA-ECMO–supported high-risk PE.

**Supplementary Information:**

The online version contains supplementary material available at https://doi.org/10.1007/s11239-026-03298-0.

## Introduction

 More than one million people were hospitalized for venous thromboembolism (VTE) in the United States in 2020, with approximately 40% of cases attributed to pulmonary embolism (PE) [1]. Acute high-risk PE is a rare condition [2], but is associated with significant mortality (39–68%) [3–7], reaching > 90% in case series of patients presenting with cardiac arrest [8].

Guideline-directed treatment of high-risk PE involves systemic anticoagulation and thrombolytic therapy [9]. Recommendations for other therapies, including catheter-directed interventions and surgical embolectomy, are varied [9, 10]. Despite the advancements in therapies, the prognosis for PE complicated by cardiac arrest and cardiogenic shock remains poor. The European Resuscitation Council Guidelines recommend extracorporeal cardiopulmonary resuscitation (ECPR) with veno-arterial extracorporeal membrane oxygenation (VA-ECMO) for high-risk PE-related cardiac arrest when conventional methods fail [11]. The principal cause of death in patients with PE is right ventricular failure. VA-ECMO rapidly stabilizes patients by decompressing the right ventricle, providing oxygenation, and maintaining systemic perfusion, breaking the self-propagating cycle of right ventricular failure [3, 12]. VA-ECMO is the preferred mechanical circulatory support for patients with massive PE given that it provides both circulatory and respiratory support and may facilitate definitive therapies such as thrombectomy [9, 13–16].

The use of VA-ECMO in the management of high-risk PE is growing at a rate of 0.07% to 1.1% from 2005 to 2013, according to national databases [17]. While existing literature suggests improved survival outcomes associated with VA-ECMO in high-risk PE, variability in clinical practice and outcomes persist. Complications, particularly significant bleeding events, frequently accompany VA-ECMO support [18, 19]. Given these considerations, our study aimed to evaluate clinical outcomes, complications, and predictors of mortality in patients treated with VA-ECMO for high-risk PE presenting with cardiac arrest or shock at a high-volume resuscitation center.

## Methods

### Study Design and Population

This retrospective cohort study was conducted at the University of Minnesota, a high-volume ECMO and resuscitation center. Consecutive adult patients (≥ 18 years) treated with VA-ECMO from January 2015 to May 2024 were screened for inclusion. Patients were included if they presented with high-risk PE defined by European Society of Cardiology (ESC) criteria: shock (systolic blood pressure < 90 mmHg, vasopressor requirement, or evidence of end-organ hypoperfusion) or cardiac arrest directly attributed to PE. Exclusion criteria included incidental or hemodynamically insignificant PE, ECMO cannulation for another indication, or PE identified following ECMO decannulation.

### Ethical Considerations

The study was conducted following the ethical principles outlined in the Declaration of Helsinki. Approval was obtained from the University of Minnesota Institutional Review Board (IRB #1703M11301), and a waiver of informed consent was granted due to the retrospective nature of the study involving minimal risk to participants.

### Data Collection

Detailed data were retrospectively extracted from electronic medical records. Collected information included patient demographics, comorbid conditions, clinical presentation (shock vs. cardiac arrest), diagnostic imaging findings (computed tomography pulmonary angiography [CTPA], pulmonary angiography, autopsy), echocardiographic parameters, laboratory data (including lactate, troponin, BNP, hemoglobin), procedural details (time from diagnosis to ECMO cannulation, types of reperfusion therapies administered such as systemic thrombolysis, catheter-directed therapy [CDT], or surgical thrombectomy), complications, post-cannulation blood transfusion requirements, ICU and hospital length of stay, and survival outcomes. We included the closest vital and laboratory data to the time of cardiac arrest or shock onset. Moreover, echocardiographic and electrocardiographic data was obtained from studies performed closest to the time of ECMO cannulation, when available. All cases were individually reviewed to confirm that PE was the primary driver of hemodynamic instability prompting VA-ECMO cannulation. In cases of uncertainty, a second physician reviewer adjudicated the diagnosis, and consensus was reached. Adjudication was not blinded to clinical outcomes, which may introduce bias in determining PE as the primary etiology.

### Definitions and Outcomes

High-risk PE diagnosis was confirmed via CTPA, pulmonary angiography, or autopsy findings. The cohort was divided into two groups defined by VA-ECMO indication, which included cardiac arrest and shock. The cardiac arrest cohort was defined as patients who experienced cardiac arrest prior to VA-ECMO cannulation, including both those cannulated during ongoing resuscitation and those who achieved return of spontaneous circulation (ROSC) but subsequently required ECMO for refractory shock. Out-of-hospital cardiac arrest was defined as cardiac arrest sustained in the pre-hospital or emergency department settings. Major bleeding following ECMO cannulation was classified according to Extracorporeal Life Support Organization (ELSO) criteria, encompassing significant hemoglobin reduction (> 2 g/dL), or transfusion requirement (≥ 2 units of packed red blood cells within 24 h) in the setting of bleeding, or bleeding at critical anatomical locations (intracranial, retroperitoneal, pulmonary, gastrointestinal, access-site, or requiring surgical intervention) [19]. End-organ hypoperfusion was defined as altered mental status, cold or mottled skin, oliguria or anuria, or increased serum lactate level. Primary outcomes were mortality rates, while secondary outcomes included major bleeding complications, length of hospital and ICU stays, and predictors of mortality.

### Statistical Analysis

Descriptive statistics summarized patient characteristics and outcomes. Univariable analyses compared demographic, clinical, imaging, procedural, and outcome variables between patients presenting with cardiac arrest versus shock and between survivors versus non-survivors. Categorical variables were analyzed using Fisher’s exact or chi-square tests, and continuous variables were compared with Student’s T-Test or Mann-Whitney U-Test as appropriate. Multivariable logistic regression analyses were performed to identify independent predictors of mortality, selecting significant variables from univariable analyses (*P*-value ≤ 0.10). Statistical significance was defined by a *P*-value < 0.05. Analyses were performed using SPSS software (IBM Corp., Version 30.0.0.0, Armonk, NY).

## Results

### Study Population and Baseline Data

**Fig. 1:**
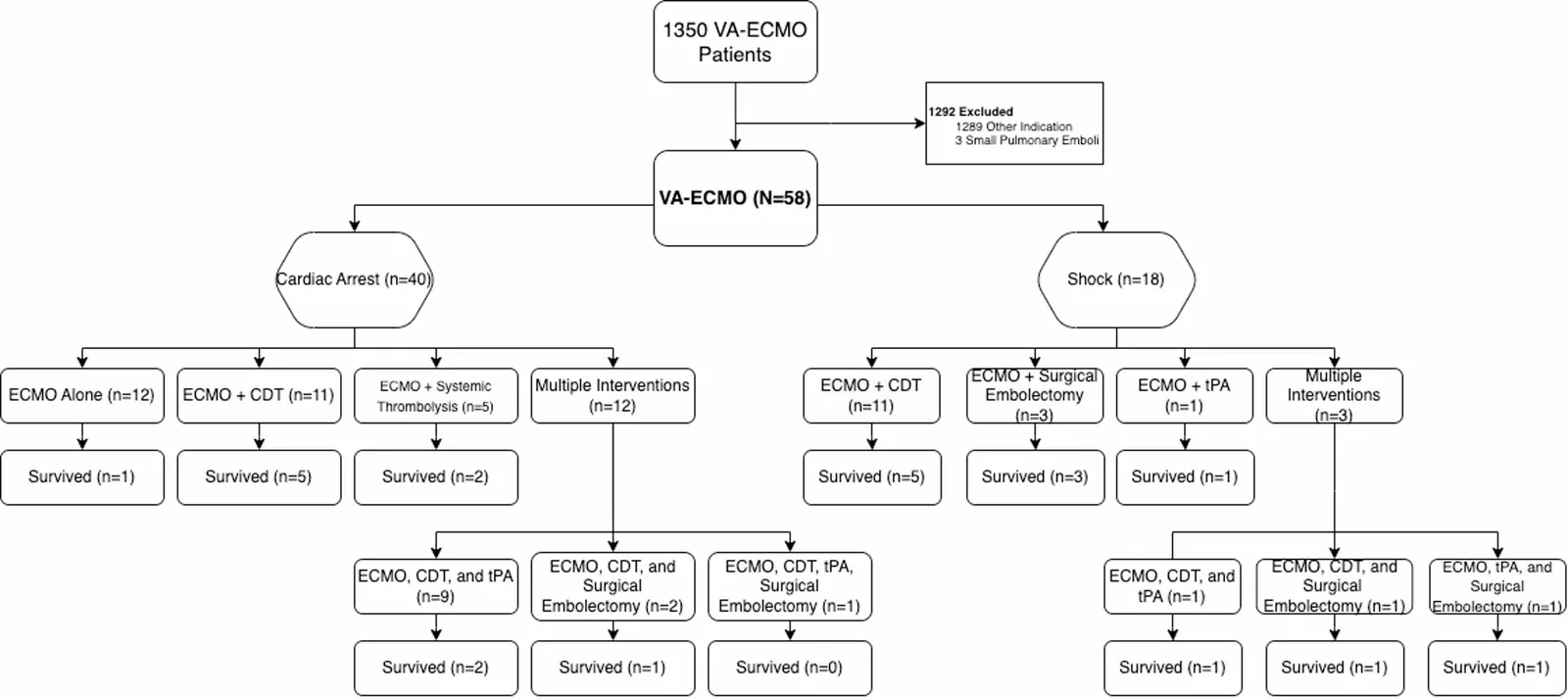
Study population organized by VA-ECMO indication and treatment

Among 1350 patients supported with VA-ECMO during the study period, 58 patients (4.3%) met the criteria for high-risk PE and were included in the analysis. Of these, 40 patients (69%) were cannulated following cardiac arrest, and 18 (31%) were cannulated for shock without cardiac arrest (Fig. 1). All patients were initiated on therapeutic systemic anticoagulation with unfractionated heparin.

The mean age of the cohort was 51.7 years (SD 14.3 years), and 55% were female (Table 1). Obesity was the most prevalent comorbidity (67.2%), followed by tobacco use (34.5%) and hypertension (27.6%). Compared to the shock group, the cardiac arrest group had higher initial lactate levels (8.5 vs. 5.0 mmol/L, *P* = 0.015; Table 2) and higher presentation Pulmonary Embolism Severity Index scores (144 vs. 112, *P* = 0.012).

**Table 1:** Baseline demographics and comparison by clinical presentation

	Entire Cohort (*N* = 58)	Cardiac Arrest (*n* = 40)	Shock (*n* = 18)	*P*-value
Age (years; ± SD)	51.7 (14.3)	52.7 (14.7)	49.4 (13.4)	0.42
Sex (Female, %)	32 (55)	22 (55)	10 (55.6)	0.97
Hypertension (%)	16 (27.6)	14 (35)	2 (11.1)	0.06
Dyslipidemia (%)	11 (19)	10 (25)	1 (9.1)	0.08
Coronary Artery Disease (%)	5 (8.6)	5 (12.5)	0	0.31
Chronic Heart Failure (%)	1 (1.7)	1 (2.5)	0	0.50
Chronic Lung Disease (%)	6 (10.3)	5 (12.5)	1 (5.6)	0.66
Chronic Liver Disease (%)	2 (3.4)	2 (5)	0	1.00
Chronic Renal Disease (%)	4 (6.9)	3 (7.5)	1 (5.6)	1.00
Obstructive Sleep Apnea (%)	8 (13.8)	4 (10)	4 (22.2)	0.21
Diabetes Mellitus (%)	8 (13.8)	6 (15)	2 (11.1)	0.69
Obesity (%)	39 (67.2)	26 (65)	13 (72.2)	0.59
BMI (± SD)	36.3 (9.4)	35.9 (10.2)	37.0 (7.7)	0.70
History of Active Cancer (%)	4 (6.9)	4 (10)	0	0.30
History of VTE (%)	16 (27.6)	8 (20)	8 (44.4)	0.054
Family History of VTE (%)	1 (1.7)	0	1 (5.6)	0.31
History of COVID-19 Infection (%)	9 (15.5)	6 (15)	3 (16.7)	0.87
History of Tobacco Use (%)	20 (34.5)	13 (32.5)	7 (38.9)	0.64
Recent Surgery or Immobilization (%)	10 (17.2)	7 (17.5)	3 (16.7)	0.94
Recent Hospitalization (%)	13 (22.4)	8 (20)	5 (27.8)	0.51

BMI, body mass index; COVID-19, coronavirus disease 2019; VTE, venous thromboembolism.

CT imaging confirmed PE in all patients within the shock cohort, whereas diagnosis in the cardiac arrest group was often made via pulmonary angiography (37.5%) or autopsy (5%) (Table 2). The cardiac arrest group demonstrated a higher incidence of ECG changes of incomplete or complete right bundle branch block (48.7% vs. 11.1%, *P* = 0.006).

**Table 2:** Comparison of clinical, laboratory, electrocardiographic, and imaging characteristics

	Cardiac Arrest (*n* = 40)	Shock (*n* = 18)	*P*-value
PESI (± SD)	144 (46.4)	112 (39.2)	0.012
sPESI (%)			
0 1 2 3 4	6 (15) 9 (22.5) 18 (45) 6 (15) 1 (2.5)	1 (5.6) 10 (55.6) 6 (33.3) 1 (5.6) 0	0.20
Initial SBP (mmHg; IQR)*	115 (34)	114 (25)	0.90
Initial DBP (mmHg; IQR)*	75 (31)	71 (14)	0.87
Initial MAP (IQR)*	89 (30)	87 (16)	0.88
Initial Heart Rate (BPM; IQR)*	111 (30)	112 (20)	0.91
Initial Lactic Acid Level (mmol/L± SD)	8.5 (5.1)	5.0 (4.3)	0.015
Initial Troponin Elevation (%)	37 (100)	17 (94.4)	0.33
Initial BNP Elevation (%)	9 (81.8)	13 (81.3)	1.00
Initial pH (± SD)	7.08 (0.27)	7.25 (0.21)	0.13
Initial Hemoglobin (g/dL; ± SD)	12.3 (2.8)	13.4 (2.1)	0.21
Diagnosis			
Computed Tomography (%) Pulmonary Angiography (%) Autopsy (%)	23 (57.5) 15 (37.5) 2 (5)	18 (100) 0 0	0.001 0.003 1.00
Deep Venous Thrombosis (%)	10 (55.6)	8 (50)	0.75
Transthoracic Echocardiogram (%) Moderate/Severe Dilatation (%) Moderate/Severe Dysfunction (%) McConnell Sign (%) Estimated PASP (mmHg; ± SD)	32 (80) 15 (48.4) 26 (83.9) 8 (25.8) 44 (15.7)	16 (88.9) 13 (81.3) 15 (93.8) 5 (35.7) 56 (12.5)	0.41 0.03 0.65 0.50 0.04
Electrocardiogram			
Sinus Tachycardia (%) Atrial Fibrillation/Flutter (%) iRBBB/RBBB (%) S1Q3T3 (%) Anterior TWI (%)	20 (51.3) 3 (7.7) 19 (48.7) 11 (28.2) 12 (30.8)	11 (61.1) 1 (5.6) 2 (11.1) 8 (44.4) 6 (33.3)	0.49 1.00 0.006 0.23 0.85

BNP, brain natriuretic peptide; BPM, beats per minute; COVID-19, coronavirus disease 2019; DBP, diastolic blood pressure; iRBBB/RBBB, incomplete right bundle branch block; MAP, mean arterial pressure; PESI, Pulmonary Embolism Severity Index; sPESI, Simplified Pulmonary Embolism Severity Index; SBP, systolic blood pressure; TWI, T-wave inversions; VTE, venous thromboembolism.

*Initial values represent the first documented measurements closest to the time of PE diagnosis or clinical deterioration. These values may reflect post-resuscitation physiology in patients with cardiac arrest or augmented values in the setting of vasopressors/inotropes.

### Intervention

In total, 45 (78%) patients had a PE diagnosed before ECMO cannulation. Time from PE diagnosis to ECMO cannulation in this population was significantly shorter in cardiac arrest patients (Table 3; 3.9 [IQR 1.4–22.7] vs. 50.9 [IQR 5.9–80.3] hours, *P* = 0.01). Use of CDT and systemic thrombolysis was similar between groups. Surgical thrombectomy was more common in the shock group (27.8% vs. 7.5%, *P* = 0.04). Patients receiving VA-ECMO alone (no additional reperfusion therapy) were more frequently from the cardiac arrest group than the shock group (30% vs. 0%, *P* = 0.009).

**Table 3:** Treatment, outcome, and complication data

	Cardiac Arrest(n=40)	Shock(n=18)	*P*-value
Catheter-Directed Treatment (CDT; %) USAT (%) Catheter-Directed Thrombolysis (%) Mechanical Thrombectomy (%)	23 (57.5) 8 (34.8) 6 (26.1) 17 (73.9)	13 (72.2) 5 (38.5) 3 (23.1) 11 (84.6)	0.29 0.83 0.84 0.46
Diagnosis to CDT (hours, ± SD)	11.6 (16.0)	28.7 (42.0)	0.18
Diagnosis to ECMO (hours, IQR)	3.9 (1.4–22.7)	50.9 (5.9–80.3)	0.01
Cannulation Configuration			
Peripheral (%) Central (%)	39 (97.5) 1 (2.5)	16 (88.9) 2 (11.1)	0.47
Systemic Thrombolysis (%)	15 (37.5)	3 (16.7)	0.11
Surgical Thrombectomy (%)	3 (7.5)	5 (27.8)	0.04
ECMO Alone (%)	12 (30)	0	0.009
Inferior Vena Cava Filter (%)	2 (5)	3 (16.7)	0.17
Intubation Duration (hours, ± SD)	175 (161)	233 (245)	0.29
Major Bleeding (%) Intracranial (%) Retroperitoneal (%) Access-site (%) Gastrointestinal (%) Pulmonary (%) Other (%)	28 (70) 9 (22.5) 11 (27.5) 8 (20) 6 (15) 6 (15) 3 (7.5)	11 (61.1) 1 (5.6) 2 (11.1) 4 (22.2) 2 (11.1) 2 (11.1) 4 (22.2)	0.51 0.11 0.17 0.85 0.69 0.69 0.11
Transfusion (%)	38 (95)	15 (83.3)	0.14
Renal Failure (%)	14 (35)	4 (22.2)	0.33
Limb Ischemia (%)	1 (2.5)	3 (16.7)	0.08
Bacteremia (%)	1 (2.5)	3 (16.7)	0.08
Gut Ischemia (%)	3 (7.5)	1 (5.6)	1.00
Vasoplegia (%)	3 (7.5)	2 (11.1)	0.64
Survival (%)	11 (27.5)	12 (66.6)	0.005
Survivor CPC			
1 or 2 (%) 3 or 4 (%)	8 (72.7) 3 (27.3)	8 (66.7) 4 (33.3)	1.00

CDT, catheter-directed therapy; CPC, cerebral performance categories; USAT, ultrasound-assisted thrombolysis.

### Survival and Complications

Overall, survival to hospital discharge was 39.7% (23/58), with higher survival among patients presenting with shock than those presenting with cardiac arrest (Tables 3 and 66.6% vs. 27.5%, *P* = 0.005). There were no significant differences in favorable neurologic outcomes (Cerebral Performance Category 1 or 2) among survivors (Table 3). A transfusion was required in 91% of the cohort. Major bleeding occurred in 67% of patients, with no significant difference between groups. Pulmonary bleeding was significantly more common in patients who did not receive systemic thrombolysis (Supplemental Table S1; *P* = 0.04). There was no significant association between mortality and major bleeding in the cardiac arrest cohort (Table 4; OR 0.83, 95% CI 0.18–3.90), and in the shock cohort (OR 5.00, 95% CI 0.44–56.62).

**Table 4:** Logistic regression analysis by clinical presentation

	Cardiac Arrest - Univariable OR (95% CI)	*P*-value	Cardiac Arrest - Multivariable OR (95% CI)	*P*-value	Shock - Univariable OR (95% CI)	*P*-value
Age (years)	1.01 (0.96–1.06)	0.74			0.91 (0.83–1.01)	0.075
Sex (Female)	0.35 (0.08–1.59)	0.17			0.25 (0.31-2.00)	0.19
PESI	1.01 (0.99–1.02)	0.54			0.98 (0.95–1.01)	0.23
iRBBB/RBBB	0.72 (0.18–2.93)	0.65				
S1Q3T3	0.58 (0.13–2.61)	0.48			4.00 (0.50-31.98)	0.19
Out-of-Hospital Cardiac Arrest	7.00 (1.48–33.21)	0.014	6.67 (1.15–38.7)	0.035		
Non-Sustained ROSC	9.33 (1.05–82.64)	0.045	8.24 (0.79-86.0)	0.078		
Duration of CPR (mins)	1.03 (0.99–1.07)	0.10	1.02 (0.99–1.06)	0.23		
Catheter-directed treatment	0.40 (0.09–1.83)	0.24				
Systemic Thrombolysis	1.07 (0.25–4.51)	0.93				
Surgical Thrombectomy	0.74 (0.06–9.09)	0.81				
ECMO Alone	6.11 (0.69–54.51)	0.11				
Major Bleeding	0.83 (0.18–3.90)	0.82			5.00 (0.44–56.62)	0.19
Time from Cardiac Arrest/Shock to ECMO	0.94 (0.22–3.94)	0.93			1.0 (0.98–1.01)	0.71

CPR, cardiopulmonary resuscitation; ECMO, Extracorporeal membrane oxygenation; iRBBB/RBBB, incomplete right bundle branch block; PESI, Pulmonary Embolism Severity Index; ROSC, return of spontaneous circulation.

Among patients with cardiac arrest, multivariable analysis identified out-of-hospital arrest (OR 6.67, 95% CI 1.15–38.7) as an independent predictor of mortality. Lack of sustained ROSC was significantly associated with survival in univariable analysis (9.33, 95% CI 1.05–82.64) but lost significance after adjustment in the multivariable model (8.24, 95% CI 0.79-86.0). In the shock cohort, no independent predictors of mortality reached statistical significance.

Among non-survivors, neurologic injury and multiorgan decompensation were the most common causes of death (Supplemental Table S2). Neurologic prognosis or brain death accounted for 41.4% of deaths in the cardiac arrest cohort and 16.7% in the shock cohort, while multiorgan failure represented 17.2% and 66.7% of deaths in each group, respectively. Additional causes included hemorrhagic shock, ischemic bowel, and in one case, early family-initiated withdrawal of care.

### Discussion

This study provides one of the largest single-center characterizations of patients treated with VA-ECMO for high-risk PE, describing the clinical features, management strategies, complications, and mortality predictors associated with this critically ill cohort. Our findings reaffirm the high mortality burden of PE-associated cardiac arrest and highlight important differences in outcomes between patients cannulated for shock versus those cannulated following cardiac arrest. While both groups fall under the ESC definition of high-risk PE, the indication for VA-ECMO differs substantially, with ECMO serving primarily as a supportive therapy in shock and as a resuscitative intervention following cardiac arrest. Accordingly, outcomes and therapeutic implications should be interpreted separately for these populations.

PE diagnosis in hemodynamically unstable patients remains challenging. While bedside imaging and clinical suspicion often guide initial resuscitation, definitive diagnostic evaluation is frequently delayed or unavailable, especially in cardiac arrest. VA-ECMO offers a critical bridge to both diagnostic clarity and therapeutic intervention in these unstable patients. In our cohort, diagnostic strategies included CTPA and invasive pulmonary angiography; however, in some cases, PE was confirmed only at autopsy.

While traditional strategies such as vasopressors, inotropes, fluid resuscitation, and low PEEP mechanical ventilation remain essential for right ventricular support [9], these approaches have limitations [9, 20–24]. VA-ECMO is an increasingly utilized support modality for high-risk PE [6, 12]; however, evidence of its benefit within this population remains mixed and mainly limited to observational studies and meta-analyses [5, 6], 25– [27].

Of our entire cohort, 23 (39.7%) survived to hospital discharge, consistent with rates reported in studies examining patients with acute PE support on VA-ECMO (39–68% mortality) [3–6]. Among patients cannulated following cardiac arrest, mortality outcomes were poor and notably higher in patients cannulated post-cardiac arrest compared to those with shock alone (72.5% vs. 33.3%, *P* = 0.005). These findings should be interpreted with caution, as outcomes in the cardiac arrest cohort reflect ECPR in PE-related arrest rather than the effectiveness of VA-ECMO as a therapeutic strategy for PE. The mortality rate in our cardiac arrest population is consistent with prior reports of ECPR across various etiologies [3, 6]. CPR may be less effective in the setting of massive PE due to impaired pulmonary outflow. This is biologically plausible, as mechanical CPR is inherently limited in the setting of obstructed pulmonary outflow, resulting in inadequate forward flow and prolonged low-perfusion states. Furthermore, most deaths after cardiac arrest are due to brain injury and multiorgan failure resulting from low-flow and hypoxemia, both of which can be avoided with timely VA-ECMO cannulation. Mortality with VA-ECMO may remain elevated due to delays in activating this therapy. Identifying the predictors of patients who develop catastrophic PE and reaching them promptly are important to improve the potential benefit of this lifesaving therapy. In contrast, patients cannulated for shock represent a distinct population in whom VA-ECMO functions primarily as a hemodynamic support strategy, allowing for right ventricular unloading, stabilization, and facilitation of definitive therapies. The substantially higher survival observed in this group suggests greater potential for benefit when ECMO is initiated prior to circulatory collapse.

Out-of-hospital cardiac arrest was a powerful predictor of mortality in the cardiac arrest cohort. In-hospital arrest, when compared to out-of-hospital arrest and shorter CPR duration (25.9 vs. 44.2 min, *P* = 0.02), were associated with higher survival. Early and effective CPR in a resource-rich hospital environment may explain these results. All patients who did not achieve ROSC (Supplemental Table S3, *n* = 9) died, suggesting prolonged low-flow states compromise outcomes. Failure to achieve sustained ROSC was a significant predictor of mortality in our univariable analysis, but not in the multivariable analysis which may be partly explained by our limited sample size. Future research should investigate the effect of time to ECMO cannulation on outcomes, especially as it is suggested that when PE is suspected as the cause of cardiac arrest, extended CPR for at least 60–90 min following systemic thrombolysis administration is advised [28].

Cause-specific mortality patterns diverged markedly between groups, with neurologic injury predominating in cardiac arrest patients and multiorgan failure more common in the shock cohort. These findings again reinforce the importance of minimizing low-flow time and optimizing perfusion strategies in cardiac arrest.

Interest in CDT has increased, especially as an adjunct to ECMO. The 2019 ESC guidelines provided a IIb recommendation for the consideration of VA-ECMO in conjunction with surgical embolectomy or catheter-directed therapies in patients with hemodynamically significant PE or those suffering cardiac arrest secondary to PE [9]. However, the literature remains mixed, likely due to heterogeneity in the CDT approach (mechanical vs. thrombolytic), timing (pre- vs. post-ECMO), and clinical presentation (shock vs. cardiac arrest) [5, 6, 27, 29]. Our cohort was too small to draw definitive conclusions, but neither CDT nor systemic thrombolysis was associated with reduced mortality. VA-ECMO alone was associated with numerically higher mortality in cardiac arrest patients, although this did not reach statistical significance (*P* = 0.08). The role of ECMO as a stand-alone therapy versus a therapeutic bridge remains uncertain [5, 19, 27, 29, 30].

Historically, clinicians have been apprehensive about the administration of systemic thrombolytics before ECMO cannulation due to bleeding concerns in the setting of large-bore access and systemic anticoagulation. Our study did not demonstrate an association between major bleeding and survival (Supplemental Table S3; 72.7% vs. 69.0%, *P* = 0.82). This may reflect intensive care practices at a specialized resuscitation center where the entire clinical team is familiar with managing patients on mechanical circulatory support. There were no differences in overall major bleeding events between patients receiving or not receiving systemic thrombolysis (Supplemental Table S2; 72.2% vs. 65.0%, *P* = 0.59). Interestingly, all pulmonary bleeding events occurred in patients who did not receive systemic thrombolysis (*n* = 8, *P* = 0.04), suggesting that processes other than systemic thrombolysis influence bleeding risks while treated with VA-ECMO. We hypothesize that a lack of timely reperfusion results in increased rates of pulmonary infarction and subsequent pulmonary bleeding. Larger population data is needed to evaluate this association. A large cohort study of 72 patients demonstrated similar bleeding risks between patients receiving thrombolysis versus no thrombolysis before VA-ECMO cannulation (61% vs. 59%, *P* = 1.00) [31]. These findings underscore that prior use of systemic thrombolytics should not preclude consideration of VA ECMO support.

Bleeding complications were common across both cohorts and occurred in 43 individuals in the entire cohort (74%). Logistic regression analysis does not demonstrate an association between mortality and major bleeding in the cardiac arrest (OR 0.83, 95% CI 0.18–3.90) and shock cohorts (OR 5.00, 95% CI 0.44–56.62). This is higher than reported in contemporary meta-analyses (14%-30%) [4, 16, 19, 32]. We reported according to the ELSO major bleeding criteria, which has been used previously in various ECMO populations [19]. The discrepancies in major bleeding rates may be explained by variations in the definition of bleeding [19, 27, 32]. Additionally, there were 10 (17.2%) intracranial bleeding events. This is higher than what has previously been reported in the literature [3, 5, 27], 31– [33]. There was no significant difference in the intracranial hemorrhage rate in patients who received thrombolytics compared to those treated without thrombolytics. One patient experienced intracranial bleeding following a recent intracranial surgery, and another developed hemorrhagic conversion from an ischemic stroke. Higher incidences of intracranial bleeding events may partly be attributable to increased surveillance, as our hospital routinely obtains head CTs on the day of cannulation and on post-cannulation day three. Additional contributors, such as timing of anticoagulation initiation or therapeutic anticoagulation goals, were not accounted for in this dataset, which may influence this outcome. Future research examining predictors of intracranial bleeding on VA-ECMO will help guide the management of these patients.

This study has several important limitations. This was a single-center, retrospective analysis, which may limit the generalizability of findings to other institutions with different patient populations or VA-ECMO protocols. Moreover, due to this study design, the temporal relationship between PE diagnosis and clinical deterioration may be variable, and causality cannot be definitively established in all cases. Additionally, attribution of hemodynamic collapse to PE was based on retrospective clinical assessment and may be subject to documentation bias. Additionally, the cardiac arrest cohort included a heterogeneous population of patients undergoing ECPR and those cannulated after return of spontaneous circulation, which may limit causal inference. The small sample size, particularly within the shock subgroup, may have limited the statistical power to detect significant associations in multivariable analyses. Timing of hemodynamic and echocardiographic measurements was not standardized and may reflect varying stages of resuscitation or ECMO support. Selection bias is inherent in studies involving VA-ECMO, as patients must survive long enough to undergo cannulation, and candidacy decisions may vary by clinician and institutional practices. Selection bias may account for variability in timing and type of reperfusion therapy or anticoagulation strategies, which could have influenced outcomes. Cause-of-death categorizations were based on clinical documentation and may have been subject to interpretation bias, particularly in cases involving withdrawal of care. Survivorship bias may influence the observed association between outcomes such as major bleeding, as patients who die early may not survive long enough to develop or be diagnosed with bleeding complications, potentially underestimating its true incidence. As this study included only patients with confirmed PE, we cannot exclude the possibility of under-ascertainment among VA-ECMO patients, particularly in cases where imaging was not performed or was nondiagnostic due to poor pulmonary artery opacification in the setting of severe right ventricular failure. Accordingly, our cohort may represent a conservatively identified population, and under-ascertainment of PE cases cannot be excluded. Additionally, our study cohort was limited to patients supported on VA-ECMO for high-risk PE. Therefore, patients supported with veno-venous ECMO were not systemically screened or included in this analysis, which may exclude an underrecognized population. Finally, neurologic outcomes were only available in survivors and not evaluated with standardized long-term follow-up tools. Future multicenter studies with larger populations and prospective designs are needed to validate these findings and optimize clinical strategies for the use of VA-ECMO in high-risk PE.

## Conclusion

In this single-center cohort of patients with high-risk PE supported with VA-ECMO, outcomes differed by indication for cannulation. Findings from the cardiac arrest cohort should be interpreted within the context of ECPR and not as evidence of ECMO efficacy for PE. Patients cannulated for shock had higher survival, while outcomes in those cannulated during cardiac arrest were consistent with prior ECPR cohorts. Among cardiac arrest patients, survival was associated with in-hospital arrest, shorter CPR duration, and sustained ROSC. No mortality differences were observed with catheter-directed therapy, systemic thrombolysis, or timing of ECMO initiation. These findings underscore the need to distinguish between ECMO as a resuscitative versus supportive strategy, and further studies are needed to define its role across these clinical scenarios.

## Electronic Supplementary Material

Below is the link to the electronic supplementary material.

Supplementary Material 1

## Data Availability

No datasets were generated or analysed during the current study.

## Abbreviations

BNP: Brain Natriuretic Peptide
BPM: Beats Per Minute
CDT: Catheter–Directed Therapy
CPC: Cerebral Performance Category
CPR: Cardiopulmonary Resuscitation
CT: Computed Tomography
CTPA: Computed Tomography Pulmonary Angiography
COVID-19: Coronavirus Disease 2019
DBP: Diastolic Blood Pressure
ECMO: Extracorporeal Membrane Oxygenation
ECPR: Extracorporeal Cardiopulmonary Resuscitation
ED: Emergency Department
ELSO: Extracorporeal Life Support Organization
ESC: European Society of Cardiology
ICU: Intensive Care Unit
iRBBB: Incomplete Right Bundle Branch Block
IQR: Interquartile Range
MAP: Mean Arterial Pressure
PASP: Pulmonary Artery Systolic Pressure
PE: Pulmonary Embolism
PEA: Pulseless Electrical Activity
PESI: Pulmonary Embolism Severity Index
RBBB: Right Bundle Branch Block
ROSC: Return of Spontaneous Circulation
SBP: Systolic Blood Pressure
sPESI: Simplified Pulmonary Embolism Severity Index
TWI: T–Wave Inversions
USAT: Ultrasound–Assisted Thrombolysis
VA-ECMO: Veno–Arterial Extracorporeal Membrane Oxygenation
VF: Ventricular Fibrillation
VT: Ventricular Tachycardia
VTE: Venous Thromboembolism
