# Supplementary material for: Outcomes of Venous-Arterial Extracorporeal Membrane Oxygenation Therapy in Acute Pulmonary Embolism: A Single-Center Experience
Source: J Thromb Thrombolysis. 2026 May 20;59(7):1739–48. doi: 10.1007/s11239-026-03298-0 (PMC13654874; doi:10.1007/s11239-026-03298-0)
Supplement: Supplementary file 1 [file 11239_2026_3298_MOESM1_ESM.docx]

**Supplemental Table S1**: Clinical Outcomes and Complications by Thrombolysis Status

|  | Systemic Thrombolysis n=18) | No Systemic Thrombolysis n=40) | *P*-value |
| --- | --- | --- | --- |
| Survival (%) | 7 (38.9) | 16 (40) | 0.94 |
| Major Bleeding (%)  Intracranial (%)  Retroperitoneal (%)  Access-site (%)  Gastrointestinal (%)  Pulmonary (%)  Other (%) | 13 (72.2)  5 (27.8)  4 (22.2)  6 (33.3)  3 (16.7)  0  3 (16.7) | 26 (65)  5 (12.5)  9 (22.5)  6 (15)  5 (12.5)  8 (20)  4 (10) | 0.59  0.15  0.98  0.11  0.67  0.04  0.47 |
| Transfusion (%) | 18 (100) | 35 (87.5) | 0.31 |

**Supplemental Table S2:** Causes Specific Mortality

| Cause of Death | Cardiac Arrest (n=29) | Shock (n=6) |
| --- | --- | --- |
| Neurologic Prognosis/Brain Death (%) | 12 (41.4) | 1 (16.7) |
| Multiorgan Decompensation (%) | 5 (17.2) | 4 (66.7) |
| Hemorrhagic Shock (%) | 1 (3.4) | 0 |
| GI/Ischemic Bowel (%) | 1 (3.4) | 1 (16.7) |
| Other (%) | 1 (3.4) | 0 |

*Abbreviations*: GI, gastrointestinal

**Supplemental Table S3**: Survival characteristics in patients presenting with cardiac arrest and shock.

|  | Cardiac Arrest (n=40) | | | Shock (n=18) | | |
| --- | --- | --- | --- | --- | --- | --- |
|  | Survivors (n=11) | Non-survivors (n=29) | *P*-value | Survivors (n=12) | Non-survivors (n=6) | *P*-value |
| Age (years; ± SD) | 51.5 (12.2) | 53.2 (15.7) | 0.75 | 53.7 (14.2) | 40.8 (5.8) | 0.052 |
| Sex (Female, %) | 8 (72.7) | 14 (48.3) | 0.17 | 8 (66.7) | 2 (33.3) | 0.32 |
| PESI (± SD) | 137 (45) | 147 (47) | 0.55 | 120 (38) | 96 (40) | 0.24 |
| iRBBB/RBBB (%) | 6 (54.5) | 13 (46.4) | 0.65 | 0 | 2 (33.3) | 0.10 |
| S1Q3T3 (%) | 4 (36.4) | 7 (24.1) | 0.48 | 4 (33.3) | 4 (66.7) | 0.32 |
| In-Hospital Cardiac Arrest (%) | 8 (72.7) | 8 (27.5) | 0.009 |  | | |
| Cardiac Arrest Outcome  ROSC (%)  Non-sustained ROSC (%)  No ROSC (%) | 10 (90.9)  0  1 (9.1) | 14 (48.3)  9 (31)  6 (20.7) | 0.04 |  |  |  |
| Duration of CPR (minutes, ± SD) | 25.9 (13) | 44.2 (32.7) | 0.02 |  |  |  |
| Catheter-directed treatment (%) | 8 (72.7) | 15 (51.7) | 0.23 | 7 (58.3) | 6 (100) | 0.11 |
| Systemic Thrombolysis (%) | 4 (36.4) | 11 (37.9) | 0.93 | 3 (25) | 0 | 0.52 |
| Surgical Thrombectomy (%) | 1 (9.1) | 2 (6.8) | 1 | 5 (41.7) | 0 | 0.11 |
| ECMO Alone (%) | 1 (9.1) | 11 (37.9) | 0.08 |  | | |
| Major Bleeding (%) | 8 (72.7) | 20 (69) | 0.82 | 6 (50) | 5 (83.3) | 0.17 |
| Time from Cardiac Arrest to ECMO  <1 Hour (%)  ≥1 Hour (%) | 4 (36.4)  7 (63.6) | 11 (37.9)  18 (62.1) | 0.93 |  | | |
| Time from Shock to ECMO  <24 Hour (%)  ≥24 Hour (%) |  | | | 9 (75)  3 (25) | 3 (50)  3 (50) | 0.34 |
| Time from Cardiac Arrest to ECMO (hours; IQR) | 2.6 (4.9) | 1.4 (4.9) | 0.74 |  | | |
| Days on ECMO (days, ± SD) | 5.3 (3.0) | 3.3 (2.9) | 0.06 | 6.6 (7.5) | 6 (8.3) | 0.62 |
| ICU LOS (days, ± SD) | 17.5 (8.8) | 6.3 (8.0) | <0.001 | 16.4 (16.6) | 20.6 (14.4) | 0.60 |
| Hospital LOS (days, ± SD) | 30.0 (11.5) | 10.9 (21.2) | 0.016 | 23.3 (17.4) | 21.1 (14.8) | 0.80 |

*Abbreviations*: CPR, cardiopulmonary resuscitation; ECMO, Extracorporeal membrane oxygenation; ICU, intensive care unit; LOS, length of stay; iRBBB/RBBB, incomplete right bundle branch block; PESI, Pulmonary Embolism Severity Index; ROSC, return of spontaneous circulation
